# Fertility preservation in breast cancer with oral progestin: is it an option? A pilot study

**DOI:** 10.31744/einstein_journal/2021AO5859

**Published:** 2021-08-09

**Authors:** Renato de Oliveira, Bárbara Gomes Maya, Mariana Bittencourt Silva Nogueira, Gabriel Seixas Conceição, Bianca Bianco, Caio Parente Barbosa

**Affiliations:** 1 Department of Community Health Centro Universitário FMABC Santo AndréSP Brazil Sexual, Reproductive Health, and Population Genetics, Department of Community Health, Centro Universitário FMABC, Santo André, SP, Brazil.

**Keywords:** Ovulation induction, Fertility preservation, Progestins, Reproductive techniques, assisted

## Abstract

**Objective:**

To compare the effectiveness of oral progestins and injectable gonadotropin-releasing hormone antagonist medication in cancer fertility preservation in patients with breast cancer.

**Methods:**

A cross-sectional study with 40 breast cancer patients submitted to cancer fertility preservation, who were divided into two groups according to histochemical analysis of progesterone receptors to define luteinizing hormone block: if positive, use of gonadotropin-releasing hormone antagonist, if negative, use of oral progestins. The mean age, medication days, antral follicle count, number of oocytes in metaphase II and the occurrence of ovarian hyperstimulation syndrome were compared.

**Results:**

A total of 20 patients both in the group using gonadotropin-releasing hormone antagonist, and in the group with oral progestins, respectively, had a mean age of 33.9 (32-35.8) and 33.8 (32-35.6) years; days of medications of 11 (9.7-12.3) and 12.8 (11.6-13.9), p=0.037; antral follicle count of 9 (7.11-12) and 8.5 (6-11.9), p=0.370; metaphase II oocyte number of 4 (2.1-9.8) and 7.5 (3.1-10), p=0.348; and ovarian hyperstimulation syndrome of 2 (10%) and 5 (25%) cases, p=0.212.

**Conclusion:**

The use of oral progestins, in spite of requiring longer treatment time, is effective in relation to the protocol with gonadotropin-releasing hormone antagonist, and offers greater comfort at a lower cost in breast cancer patients with negative progesterone receptors, submitted to cancer fertility preservation.

## INTRODUCTION

In Brazil, neoplasms are the second leading cause of death among women, with an incidence of 420 thousand cases per year in 2018-2019, excluding non-melanoma skin cancer.^( 1 )^ The significant incidence of cancer cases in Brazilian women, in the context of public health, requires investments, research and constant formulation of new treatment strategies for all therapeutic options.^( 2 )^

Despite the increasing survival rates in this population provided by the development of cancer treatments, the greatest concern are the late effects on quality of life.^( 2 , 3 )^ Among these, the deleterious effect on the patients’ reproductive potential stands out.^( 4 )^ Therefore, the professionals involved in the treatment are encouraged to offer guidance to female patients with cancer, as early as possible, on cancer fertility preservation (FP) options, especially if gonadotoxic treatments are indicated.^( 5 )^ The high prevalence of premature ovarian failure and infertility after chemotherapy treatments justifies cryopreservation of oocytes and embryos as a reproductive alternative.^( 6 - 8 )^

In this context, during controlled ovarian stimulation (COS), it is common to observe a supraphysiological estrogen level — which is inadvisable in estrogen-dependent cancers. Therefore, an aromatase inhibitor is associated as an adjuvant therapy to the use of gonadotropins.^( 9 , 10 )^The aromatase inhibitor reduces plasma levels of estrogen by the inhibition of conversion of androgens to estrogen. Additionally, it maintains positive feedback on the endogenous release of follicle stimulating hormone (FSH).^( 11 )^

Another effect of COS is the prevention of luteinizing hormone (LH) peak, avoiding premature ovulation, which is one of the major causes of cycle cancellations.^( 12 - 15 )^Traditionally, both gonadotropin-releasing hormone agonist (ag-GnRH) and gonadotrophin-releasing hormone antagonist (ant-GnRH) have been used as injectable medications.^( 13 )^ In cases of preservation of fertility, especially in cancer patients, the ant-GnRH protocol is generally used. Toftager et al., performed a meta-analysis with similar results between the ant-GnRH and ag-GnRH protocols in relation to live birth rates.^( 14 )^ However, a new COS protocol using oral progestin, to block the LH peak, has promising results, including in poor responders, with embryonic characteristics and pregnancy rates similar to those of ag-GnRH protocols after the transfer of frozen-thawed embryos.^( 12 , 15 , 16 )^

Regarding financial costs, oral progestins, despite their use throughout the entire COS, have a significantly lower cost compared to injectable medications, such as ag-GnRH and ant-GnRH.^( 17 )^Considering an average of 4 days of ant-GnRH use, this reduction could be as great as eight-fold lower, which means savings of approximately US$300.00.^( 18 )^

However, to date, there are no reports on the effectiveness of the use of oral progestin in FP cancer patients.

## OBJECTIVE

To compare the effectiveness of oral progestins and injectable gonadotropin-releasing hormone antagonist medication in preserving fertility in breast cancer patients.

## METHODS

This was a cross-sectional study conducted in a convenience sample of breast cancer patients in FP. Data were collected from electronic medical records of the outpatient clinic of the Human Reproduction Department of the *Faculdade de Medicina do ABC* , in Santo André (SP), in the period from November 2018 to August 2019.

The patients were divided into two groups, according to the immunohistochemical result of the progesterone receptors testing for breast cancer: if positive, ant-GnRH treatment was used, comprising the Control Group; if negative, oral progestin treatment was used, comprising the Progestin Group.

The inclusion factors were patients with breast cancer who underwent assisted reproduction technologies (ART), with the use of 200IU a day of recombinant FSH (rFSH) associated with both oral progestin and ant-GnRH variable protocols.

Exclusion factors were use of hormonal contraceptives in the last 3 months preceding treatment, previous ovarian surgery and previous chemotherapy or radiotherapy.

Clinical characteristics were age at start of treatment, age at menarche, menstrual cycles characteristics (regularity, duration, and interval between cycles), antral follicle count (AFC), number of days of medication, presence of ovarian hyperstimulation syndrome (OHSS), number of preovulatory follicles (considered in this study as greater than 14mm), number of retrieved oocytes and number of metaphase II oocytes.

The Control Group underwent COS with rFSH 200IU a day, associated with 2.5mg letrozole, two tablets a day (Femara^®^, Novartis, Switzerland), in estrogen-receptor-positive cases, and the medication was started on the second or third day of the menstrual cycle. Monitoring was carried out by conventional two-dimensional transvaginal ultrasound at 7Mhz (Philips HD 7^®^, Netherlands). AFC was performed on each ovary, and antral follicles were those sized 2 to 10mm.^( 19 , 20 )^ Hypothalamic blockade with ant-GnRH (0.25mg cetrorelix acetate, Orgalutran^-^MSD, United States of America, or Cetrotide^®^ - Serono, Germany) occurred when the largest growing follicle reached 14mm in diameter, characterizing the variable protocol. Highly purified lyophilic injectable human chorionic gonadotropin (hCG) (Choriomon^®^-M 5000IU; Meizler UCB Biopharma, Belgium) was administered when the largest follicles reached 18mm to 22mm in diameter. Ultrasonography-guided follicular aspiration for oocyte uptake was performed 35 hours after hCG injection.

The Progestin Group also received 200IU of rFSH daily, together with two medroxyprogesterone acetate tablets (Provera^®^ 10mg/tablet; Pfizer, USA) or two dydrogesterone tablets (Duphaston^®^ 10mg; Abbott) once a day, orally, until the triggering of ovulation with 2mL of ag-GnRH (triptorelin acetate, Gonapeptyl^®^ 0.1mg daily; Ferring, Switzerland), when the largest follicles reached between 18mm and 22mm in diameter. After 35 hours, ovarian puncture was performed.

Regarding COS, OHSS was classified as mild if there were abdominal distension and discomfort, possibly associated with nausea, vomiting and/or diarrhea, with ovarian enlargement from 5cm to 12cm; moderate, if there were features of mild OHSS plus ultrasonographic evidence of ascites; and severe, if it was associated with clinical ascites and/or hydrothorax, dyspnea, hemoconcentration, hypercoagulability, and impaired renal function.^( 21 )^

According to the degree of nuclear maturation, oocytes were classified as metaphase I or metaphase II.

Qualitative variables were expressed as absolute and relative frequencies and quantitative variables as means and medians, 25^th^ and 75^th^ percentiles, confidence interval (CI) and Student´s *t* test. To analyze the association of qualitative variables, the χ^2^ test was used; and for quantitative variables, due to the non-normal distribution of data (Shapiro-Wilk, p<0.05), the Mann-Whitney test was used. The confidence interval was 95%. The Stata 11.0 statistical software was used.

The study was approved by the Ethics Committee of the *Faculdade de Medicina do ABC* (CAAE: 90584718.8.0000.0082 and opinion number 3.076.655), and all participants signed the Informed Consent Form.

## RESULTS

A total of 40 medical records were analyzed, proportionally divided into groups of 20 patients each.

In the analysis of quantitative variables with normal distribution, data on the patients’ age, age at menarche and cycle interval, and qualitative variables on the regularity of menstrual cycles are shown in table 1 .

**Table 1:** Clinical characteristics of the studied groups

Characteristics	Control Group (GnRH antagonist)	Progestin Group (oral progestin)	p value
Age, years	33.9 (32-35.8)	33.8 (32-35.6)	0.936*
Menarche, years	12.2 (11.1-13.2)	12.1 (11.1-13.1)	0.908*
Cycle interval	28 (28-28)	28 (27.7-30)	0.945^†^
Cycles			1.000^‡^
Regulares	19 (95)	19 (95)	
Irregulares	1 (5)	1 (5)	

Results expressed as mean (95% confidence interval) or n (%).

* Student´s *t* test; ^†^ Mann-Whitney test; ^‡^ χ^2^ test.

GnRH: gonadotropin releasing hormone.

Table 2 shows data on treatment of patients undergoing cancer FP with ant-GnRH and oral progestin protocols. Aspects related to COS, such as number of days of medication, noting that both groups used a dose of 200IU of rFSH, AFC, number of preovulatory follicles, and progression to OHSS, as well as oocyte quality – metaphase I and metaphase II – and the number of oocytes retrieved on the day of the ovarian puncture were compared with the appropriate statistical test.

**Table 2:** Evaluation of controlled ovarian stimulation and laboratory data from assisted reproduction treatment

Characteristics	Control Group (GnRH antagonist)	Progestin Group (oral progestin)	p value
Days of medication	11 (9.7-12.3)	12.8 (11.6-13.9)	0.037*
AFC	9 (7.11-12)	8.5 (6-11.9)	0.370^†^
Preovulatory follicles	6 (4-13.9)	10 (4.1-15)	0.584^†^
Retrieved oocytes	4.5 (3-10.7)	9 (4.1-12.8)	0.265^†^
Metaphase I	0.5 (0-1)	0	0.113^†^
Metaphase II	4 (2.1-9.8)	7.5 (3.1-10)	0.348^†^
OHSS, n (%)			0.212^‡^
No	18 (90)	15 (75)	
Yes	2 (10)	5 (25)	

Results expressed as mean (95% confidence interval), median (95% confidence interval), or n (%).

* Student´s *t* test; ^†^ Mann-Whitney test; ^‡^ χ^2^ test.

GnRH: gonadotropin releasing hormone; AFC: antral follicle count; OHSS: ovarian hyperstimulation syndrome.

## DISCUSSION

To our knowledge, this is the first study using oral progestin to block LH peak during COS in cancer fertility preservation. Its importance consists both in the cost reduction provided by this protocol, of approximately US$300.00, when compared to traditional protocols with the use of ant-GnRH,^( 18 )^ and in the ease of administration of an oral medication when compared to an injectable medication.^( 22 )^

Considering the suffering inherent in cancer treatment, minimizing the discomfort in drug application is part of good practices for the humanization of healthcare, aiming to improve quality of life. This is defined by the World Health Organization (WHO) as “the individuals’ perception of their position in life in the context of the culture and value systems in which they live and in relation to their goals, expectations, standards and concerns.”^( 23 )^ It is a multifaceted concept, affected by the individuals’ physical health, psychological status, personal beliefs, social relationships and their relation with important characteristics of their environment.^( 23 )^

Oocyte cryopreservation is indicated for cancer FP cases, because it increases the chance of their future use, for social reasons arising from a possible marital separation during cancer treatment, and because it expands the possibilities for patients without partners or with restrictions on the use of donated semen. In addition, it minimizes religious or ethical objections to freezing embryos.^( 24 )^

Estrogen receptor positive patients received letrozole, aiming to reduce exposure to the total amount and peak estrogen levels during cancer FP. In these scenarios, Hussein et al., demonstrated that the use of letrozole in patients with breast cancer does not result in a short-term worsening of survival or an increase in the cancer recurrence rate at the 5-year follow-up. Additionally, letrozole COS in breast cancer patients undergoing FP has pregnancy rates comparable to results from conventional COS protocols in cancer-free controls.^( 25 )^ Therefore, it is suggested the use of letrozole in our study has not impacted the results shown.

Kuang et al., started comparative studies on the use of an oral progestin, medroxyprogesterone acetate, with ant-GnRH in patients undergoing ART. Despite the increased number of retrieved oocytes, and the mean duration of treatment days with oral medication, there was no statistically significant difference.^( 12 )^ In the present study, although the median number of oocytes in the group using oral progestin was also higher, there was no statistically significant difference. On the other hand, the mean number of days of treatment with the use of oral progestin was higher (12.8 days) when compared to 11 days in the Control Group (p=0.037). Possible explanations can be related to the small size of the study sample and the tendency to avoid scheduling puncture procedures on weekends, for the convenience of the physician, and because of higher operational labor costs on weekends in Brazil. Despite the lack of a thorough cost assessment, a trend towards a small extension of the cycle is suggested, when convenient, considering the lower cost of oral progestin when compared to injectable ant-GnRH.

In the case of OHSS risk, the use of oral medroxyprogesterone acetate was compared to the use of ant-GnRH in patients with polycystic ovary syndrome in a prospective controlled study. Despite the absence of OHSS cases in the Progestin Group, there were two cases in the Control Group, with no statistically significant difference.^( 26 )^ Barbosa et al., reported a 5% prevalence of OHSS in Brazilian patients who used 200IU per day of rFSH associated with ant-GnRH.^( 19 )^ Induction of ovulation also deserves consideration. The use of an ag-GnRH analogue, namely triptorrelin acetate, can be used both in antagonist stimulation protocols, instead of the conventional hCG,^( 25 )^ and in COS protocols with oral progestin, with good response in oocyte quality.^( 27 )^ Ag-GnRH has a shorter half-life and a lower incidence of OHSS when compared to hCG to induce ovulation. Avoiding OHSS is crucial in cancer FP, due to the risk of postponing oncological treatment.^( 25 )^ In our study, OHSS occurred in five out of 20 cases (25%), and two out of 20 cases (10%), respectively, in the progestin and control groups, with p=0.212. The small number of participants limits a comparison of these data with those frequently reported in the literature. However, this higher percentage of OHSS incidence could be related to the fact that many patients were young, with good ovarian reserve, and received standardized and unadjusted doses of rFSH, due to the limited time available for cancer FP, aiming to increase the number of cryopreserved oocytes and, consequently, the reproductive chances. The absence of ascites cases stands out, and all cases were classified as mild OHSS.

Another oral progestin, dydrogesterone, was compared to medroxyprogesterone acetate in a prospective randomized controlled trial. There was no statistically significant difference in the number of oocytes, embryos, and pregnancy rate. Additionally, dydrogesterone effectively inhibited the premature increase in LH without impacting serum progesterone values.^( 28 )^ This justified the inclusion of the use of both medroxyprogesterone acetate and dydrogesterone in the group using oral progesterone. Due to the small convenience sample, it was decided not to separate the patients by type of oral medication in the statistical analysis.

In this context of good effectiveness in the use of oral progestin in oocyte and embryo quality, Massin recommended its use in non-oncological fertility preservation, considering the improvements in embryo cryopreservation technique, and avoiding the immediate transfer of embryos into a endometrium with potentially defective endometrial receptivity.^( 27 )^

Similarly, La Marca et al., reinforced the idea of safety and effectiveness of oral progestins during COS, considering them a good alternative to avoid early luteinization, with good results in oocyte and embryonic number and quality, in addition to low risk of OHSS. Although they also did not mention its use in cancer FP, they highlighted the need for more studies on long-term reproductive, obstetric and neonatal outcomes in other scenarios.^( 29 )^

However, when considering progesterone receptor-negative breast cancer patients, and patients with other neoplasms, such as endometrial cancer, in which progestin has a protective effect,^( 30 )^the following question may be asked: why not use oral progestin in selected cases of cancer FP? After all, it is a medication that has been shown to be effective, with lower cost and greater comfort when compared to a second injectable medication during COS, and no evident mechanisms of action that could worsen the cancer prognosis. Furthermore, the short period of use during COS is also a motivating factor for its use.

The small number of patients is a bias in this convenience sample pilot study. Consequently, the generalizability of the data is limited. However, the strict criteria for selecting a sample of progesterone receptor-negative patients in the immunohistochemical evaluation without the use of hormonal contraception in the 3 months preceding the treatment provided an exclusive group that justified this study. The homogeneity of the studied groups and the result of more than 80% of mature oocytes ready to be cryopreserved are factors that encourage further studies to establish the safety and effectiveness of progestin protocols in patients with cancer.

Another limiting factor was the lack of hormonal evaluation during COS cycles. The limitations inherent to a convenience sample and the limited time to start chemotherapy, to avoid delay in the treatment of cancer, allowed the beginning of COS to occur, in some cases, in the first medical assessment, preventing a hormonal evaluation from the beginning of the cycle. However, the clinical and laboratory data from a select group of this study are reinforced.

Therefore, cancer FP with oral progestin is a viable option, which could be explored in non-inferiority studies, not only to increase therapeutic strategies, but also clinical experience, aiming at cost reduction, a crucial factor for the expansion of fertility preservation programs within the perspective of a more humanized treatment.

## CONCLUSION

The use of oral progestin, despite requiring longer treatment time, is effective in relation to the gonadotropin-releasing hormone antagonist protocol and offers greater comfort at a lower cost in progesterone receptor negative breast cancer patients undergoing cancer fertility preservation.
