# Supplementary material for: Fertility preservation in breast cancer with oral progestin: is it an option? A pilot study
Source: Einstein (Sao Paulo). 2021 Aug 9;19:eAO5859. doi: 10.31744/einstein_journal/2021AO5859 (PMC8354590; doi:10.31744/einstein_journal/2021AO5859)
Supplement: Translation

# Translation: Preservação da fertilidade com progestágeno oral em pacientes com câncer de mama: é uma opção? Estudo-piloto

**Authors:** Renato de Oliveira, Bárbara Gomes Maya, Mariana Bittencourt Silva Nogueira, Gabriel Seixas Conceição, Bianca Bianco, Caio Parente Barbosa

**Affiliations:** 1 Brazil Sexual, Reproductive Health, and Population Genetics, Department of Community Health, Centro Universitário FMABC, Santo André, SP, Brazil.

**Keywords:** Indução da ovulação, Preservação da fertilidade, Progestinas, Técnicas de reprodução assistida

## Abstract

**Objetivo:**

Comparar a efetividade dos progestágenos orais em relação à medicação injetável antagonista de hormônio liberador de gonadotrofina na preservação da fertilidade oncológica de pacientes com câncer de mama.

**Métodos:**

Estudo transversal com 40 pacientes com câncer de mama submetidas à preservação da fertilidade oncológica, que foram divididas em dois grupos, conforme análise histoquímica dos receptores de progesterona, para definir o bloqueio de hormônio luteinizante: se positivos, uso de antagonista de hormônio liberador de gonadotrofina; se negativos, uso de progestágenos orais. Comparou-se média de idade, dias de medicações, contagem de folículos antrais, número de oócitos em metáfase II e ocorrência de síndrome do hiperestímulo ovariano.

**Resultados:**

Vinte pacientes, tanto no grupo com uso de antagonista de hormônio liberador de gonadotrofina, quanto no grupo com progestágenos orais, respectivamente, apresentaram média de idade de 33,9 (32-35,8) e 33,8 (32-35,6) anos; 11 (9,7-12,3) e 12,8 (11,6-13,9) de dias de medicações com p=0,037; contagem de folículos antrais de 9 (7,11-12) e 8,5 (6-11,9), com p=0,370; número de oócitos metáfase II de 4 (2,1-9,8) e 7,5 (3,1-10), com p=0,348, e síndrome do hiperestímulo ovariano de 2 casos (10%) e 5 casos (25%), com p=0,212.

**Conclusão:**

O uso de progestágenos orais, apesar de necessitar de maior tempo de tratamento, é efetivo em relação ao protocolo com antagonista de hormônio liberador de gonadotrofina e oferece maior conforto com menor custo em pacientes com câncer de mama com receptores de progesterona negativos, submetidas à preservação da fertilidade oncológica.

## INTRODUÇÃO

No Brasil, as neoplasias se configuram como a segunda causa de óbito entre as mulheres, com incidência de 420 mil casos por ano, para o biênio 2018-2019, excetuando-se o câncer de pele não melanoma.^( 1 )^A expressiva incidência de casos de câncer em mulheres brasileiras, no âmbito da saúde pública, requer investimentos, pesquisas e elaboração constante de estratégias em todas as opções terapêuticas.^( 2 )^

Apesar de a evolução dos tratamentos oncológicos propiciar um aumento nas taxas de sobrevida dessa população, há maior preocupação acerca das repercussões tardias na qualidade de vida.^( 2 , 3 )^Dentre estas, destaca-se o efeito deletério no potencial reprodutivo.^( 4 )^Assim, há um estímulo para os profissionais envolvidos realizarem, o mais precocemente possível, orientações sobre as opções de preservação da fertilidade oncológica (PFO), principalmente se indicados tratamentos gonatóxicos.^( 5 )^A alta prevalência de insuficiência ovariana prematura e de infertilidade, após tratamentos quimioterápicos, justifica a criopreservação de oócitos e embriões como alternativa reprodutiva.^( 6 - 8 )^

Nesse contexto, é comum, durante a estimulação ovariana controlada (EOC), a ocorrência de um nível suprafisiológico estrogênico – algo desaconselhável em cânceres estrogênio-dependentes. Para tanto, associa-se um inibidor de aromatase como terapia adjuvante ao uso das gonadotrofinas.^( 9 , 10 )^O inibidor de aromatase reduz as concentrações plasmáticas de estrogênio devido à inibição da conversão de andrôgenios em estrógenos. Adicionalmente, mantém o *feedback* positivo na liberação endógena do hormônio folículo estimulante (FSH).^( 11 )^

Outro ponto da EOC é a prevenção do pico do hormônio luteinizante (LH), evitando a ovulação precoce, uma das principais causas de cancelamentos de ciclos.^( 12 - 15 )^Tradicionalmente, utilizam-se tanto medicações injetáveis de agonista do hormônio liberador de gonadotrofina (ag-GnRH), quanto de antagonista do hormônio liberador de gonadotrofina (ant-GnRH).^( 13 )^No caso da preservação da fertilidade, em especial da PFO, geralmente, utiliza-se o protocolo com ant-GnRH. Toftager et al. realizaram metanálise apresentando resultados semelhantes entre os protocolos com ant-GnRH e ag-GnRH em relação à taxa de nascidos vivos.^( 14 )^Entretanto, um novo protocolo de EOC com uso de progestágeno oral, a fim de bloquear o pico do LH, demonstrou resultados promissores, inclusive em más respondedoras, evidenciando características embrionárias e taxas de gravidezes semelhantes às de protocolos com uso de ag-GnRH após a transferência de embriões descongelados.^( 12 , 15 , 16 )^

Em relação aos custos financeiros, os progestágenos orais, apesar de seu uso ao longo de toda EOC, possuem custo significantemente menor em relação às medicações injetáveis, tanto de ag-GnRH quanto de ant-GnRH.^( 17 )^Considerando média de 4 dias de ant-GnRH, essa redução poderia chegar a oito vezes, sendo compatível com economia, aproximada, de 300 dólares americanos.^( 18 )^

Porém, até o presente momento, não há relatos sobre a efetividade do uso de progestágeno oral em pacientes em PFO.

## OBJETIVO

Comparar a efetividade dos progestágenos orais em relação à medicação injetável antagonista de hormônio liberador de gonadotrofina na preservação da fertilidade oncológica de pacientes com câncer de mama.

## MÉTODOS

Estudo transversal com amostra por conveniência de pacientes com câncer de mama em PFO. Os dados foram coletados de prontuários eletrônicos do ambulatório do setor de Reprodução Humana da Faculdade de Medicina do ABC, em Santo André (SP), no período entre novembro de 2018 até agosto de 2019.

As pacientes foram divididas em dois grupos, conforme resultado imunoistoquímico dos receptores de progestágeno da neoplasia de mama: se positivo, o tratamento utilizou ant-GnRH, formando-se o Grupo Controle; se negativo, o tratamento utilizou progestágeno oral, compondo o Grupo Progestágeno.

Os fatores de inclusão foram pacientes com câncer de mama submetidas às técnicas de reprodução assistida (TRA), com uso de 200UI ao dia de FSH recombinante (FSHr) associado tanto aos protocolos variáveis com ant-GnRH, quanto aos protocolos com progestágeno oral.

Os fatores de exclusão foram uso de contraceptivos hormonais nos últimos 3 meses precedentes ao tratamento, cirurgia ovariana prévia e quimioterapia ou radioterapia prévia.

As características clínicas foram idade no início do tratamento, idade da menarca, características dos ciclos menstruais (regularidade, duração, e intervalo entre os ciclos), contagem de folículos antrais (CFA), número de dias de medicação, presença de síndrome do hiperestímulo ovariano (SHO), número de folículos pré-ovulatórios (considerado neste estudo como maiores que 14mm), número de oócitos recuperados e número de oócitos em metáfase II.

O Grupo Controle foi submetido à EOC com FSHr 200UI ao dia, associado a letrozol 2,5mg, dois comprimidos ao dia (Femara^®^, Novartis, Suiça), nos casos de receptores positivos para estrogênio, iniciado no segundo ou terceiro dia do ciclo menstrual. A monitorização ocorreu por ultrassonografia transvaginal bidimensional convencional a 7Mhz (Philips HD 7^®^, Holanda). A CFA foi realizada em cada ovário, e foram considerados folículos antrais aqueles com tamanhos de 2 até 10mm.^( 19 , 20 )^O bloqueio hipotalâmico com ant-GnRH (acetato de cetrorrelix 0,25mg, Orgalutran^-^MSD, Estados Unidos da América, ou Cetrotide^®^- Serono, Alemanha) ocorreu quando o maior folículo em crescimento alcançasse 14mm de diâmetro, caracterizando o protocolo variável. Administrou-se a gonadotrofina coriônica humana (hCG) altamente purificada liófilo injetável (Choriomon^®^-M 5000UI; Meizler UCB Biopharma, Bélgica) quando os maiores folículos atingissem entre 18mm e 22mm de diâmetro. A aspiração folicular para captação oócitária guiada por ultrassonografia foi realizada 35 horas após a injeção da hCG.

O Grupo Progestágeno também recebeu 200UI de FSHr ao dia, concomitantemente aos dois comprimidos de acetato de medroxiprogesterona (Provera^®^10mg/comprimido; Pfizer, Estados Unidos) ou dois comprimidos de didrogesterona (Duphaston^®^10mg; Abbott) uma vez por dia, via oral, até o desencadeamento da ovulação com 2mL do ag-GnRH (acetato de triptorrelina, Gonapeptyl^®^Daily 0,1mg; Ferring, Suíça), quando os maiores folículos atingissem entre 18mm e 22mm. Após 35 horas, realizou-se a punção ovariana.

Em relação à EOC, considerou-se SHO leve se distensão e desconforto abdominal, possivelmente associados a náuseas, vômitos e/ou diarreias e ovários aumentados para 5cm a 12cm; moderada, se características de SHO leve mais evidência ultrassonográfica de ascite, e grave, se possível associação com ascite clínica e/ou hidrotórax, dispneia, hemoconcentração, hipercoagulabilidade e alteração da função renal.^( 21 )^

De acordo com o grau de maturação nuclear, os oócitos foram classificados em metáfase I ou metáfase II.

As variáveis qualitativas foram apresentas por frequências absoluta e relativa e as quantitativas por médias e medianas, percentis 25 e 75, intervalo de confiança (IC) e teste *t* de *Student* . Para analisar associação entre variáveis qualitativas, utilizou-se teste do χ^2^; e, para as quantitativas, devido à não distribuição normal dos dados (Shapiro-Wilk, p<0,05), utilizou-se teste de Mann-Whitney. O intervalo de confiança foi 95%. Foi utilizado programa estatístico Stata 11.0.

O estudo foi aprovado pelo comitê de ética da Faculdade de Medicina do ABC (CAAE: 90584718.8.0000.0082 e número de parecer 3.076.655), e todas as participantes assinaram o Termo de Consentimento Livre e Esclarecido.

## RESULTADOS

Foram analisados 40 prontuários com divisão proporcional de 20 pacientes para cada grupo.

Na análise das variáveis quantitativas com distribuição normal, tanto os dados em relação à idade das pacientes, idade da menarca e intervalo dos ciclos, quanto as variáveis qualitativas em relação à regularidade dos ciclos menstruais estão apresentados na tabela 1 .

**Tabela 1:** Características clínicas dos grupos estudados

Características	Grupo Controle (antagonista GnRH)	Grupo Progestina (progestágeno oral)	Valor de p
Idade, anos	33,9 (32-35,8)	33,8 (32-35,6)	0,936*
Menarca, anos	12,2 (11,1-13,2)	12,1 (11,1-13,1)	0,908*
Intervalo dos ciclos	28 (28-28)	28 (27,7-30)	0,945^†^
Ciclos			1,000^‡^
Regulares	19 (95)	19 (95)	
Irregulares	1 (5)	1 (5)	

Resultados expressos por média (intervalo de confiança de 95%) ou n (%).

* teste *t* de *Student* ;^†^teste de Mann-Whitney;^‡^teste do χ^2^.

GnRH: hormônio liberador de gonadotrofina.

A tabela 2 apresenta dados relacionados ao tratamento das pacientes submetidas à PFO com protocolos com uso de ant-GnRH e com uso de progestágeno oral. Aspectos relacionados com a EOC, como número de dias de medicações, ressaltando que ambos os grupos utilizaram a dose de 200UI de FSHr, a CFA, o número de folículos pré-ovulatórios, e a evolução para SHO, assim como a qualidade oocitária – metáfase I e metáfase II – e o número de oócitos recuperados no dia da punção ovariana, foram comparados com o teste estatístico adequado.

**Tabela 2:** Avaliação da estimulação ovariana controlada e dos dados laboratoriais do tratamento de reprodução assistida

Características	Grupo Controle (antagonista GnRH)	Grupo Progestina (progestágeno oral)	Valor de p
Dias de medicação	11 (9,7-12,3)	12,8 (11,6-13,9)	0,037*
CFA	9 (7,11-12)	8,5 (6-11,9)	0,370^†^
Folículos pré-ovulatórios	6 (4-13,9)	10 (4,1-15)	0,584^†^
Oócitos recuperados	4,5 (3-10,7)	9 (4,1-12,8)	0,265^†^
Metáfase I	0,5 (0-1)	0	0,113^†^
Metáfase II	4 (2,1-9,8)	7,5 (3,1-10)	0,348^†^
SHO, n (%)			0,212^‡^
Não	18 (90)	15 (75)	
Sim	2 (10)	5 (25)	

Resultados expressos por média (intervalo de confiança de 95%), mediana (intervalo de confiança de 95%) ou n (%).

* teste *t* de *Student* ;^†^teste Mann-Whitney;^‡^teste do χ^2^.

GnRH: hormônio liberador de gonadotrofina; CFA: contagem de folículos antrais; SHO: síndrome do hiperestímulo ovariano.

## DISCUSSÃO

Este estudo, até o atual conhecimento, é o primeiro a utilizar progestágeno oral para o bloqueio do pico de LH durante a EOC na preservação oncológica da fertilidade. Sua importância consiste tanto na redução de custos proporcionada por este protocolo, de aproximadamente 300 dólares americanos em relação aos tradicionais protocolos com uso de ant-GnRH,^( 18 )^quanto na facilidade de administração de uma medicação via oral em relação à injetável.^( 22 )^

Considerando o sofrimento inerente ao tratamento oncológico, minimizar o desconforto na aplicação medicamentosa faz parte das boas práticas de humanização no tratamento, visando à melhoria da qualidade de vida. Esta é definida pela Organização Mundial da Saúde (OMS) como “a percepção do indivíduo de sua posição na vida no contexto da cultura e dos sistemas de valores nos quais ele vive e em relação aos seus objetivos, expectativas, padrões e preocupações”.^( 23 )^É um conceito multifacetado, afetado pela saúde física da pessoa, pelo estado psicológico, pelas crenças pessoais, pelas relações sociais e sua relação com características importantes de seu ambiente.^( 23 )^

A criopreservação de oócitos é a opção indicada para casos de PFO, tanto por aumentar a chance de seu uso futuro, por questões sociais decorrentes de uma possível separação conjugal durante o tratamento oncológico, quanto por ampliar as possibilidades para pacientes sem parceiros ou com restrições ao uso de sêmen doado. Além disso, minimiza objeções religiosas ou éticas ao congelamento de embriões.^( 24 )^

As pacientes que apresentaram positividade para os receptores estrogênicos receberam letrozol, visando à redução da exposição à quantidade total e ao pico de estrogênio durante a PFO. Nessas situações, Hussein et al., demonstraram que o uso de letrozol em pacientes com câncer de mama não resulta em piora de sobrevida a curto prazo ou em aumento na taxa de recorrência do câncer no acompanhamento em 5 anos. Adicionalmente, a EOC com letrozol, em pacientes com câncer de mama submetidas à PFO, apresenta taxas de gravidezes comparáveis às de resultados dos protocolos de EOC convencionais em controles sem câncer.^( 25 )^Assim, sugere-se que o uso de letrozol no presente estudo não tenha impactado os resultados apresentados.

Kuang et al., iniciaram os estudos comparativos do progestágeno oral acetato de medroxiprogesterona com o ant-GnRH em pacientes submetidas às TRA. Apesar do aumento no número de oócitos recuperados, e na duração média de dias de tratamento com a medicacão oral, não houve diferença estatisticamente significante.^( 12 )^No presente estudo, embora também haja maior valor na mediana do número de oócitos no grupo que usou progestágeno oral, não houve diferença estatisticamente significante. Por outro lado, a média de dias de tratamento com o uso de progestágeno oral foi superior (12,8 dias) em comparação aos 11 dias no Grupo Controle (p=0,037). Possíveis explicações podem se relacionar à restrita amostra do estudo e à tendência em evitar os finais de semana para agendamento de punção, tanto por uma maior comodidade médica, quanto pelos maiores custos operacionais trabalhistas nos finais de semana no Brasil. Apesar da não realização de uma criteriosa avaliação de custo, aventa-se a tendência, quando conveniente, em um pequeno prolongamento do ciclo, considerando o valor reduzido do progestágeno oral em relação ao injetável ant-GnRH.

No caso do risco de SHO, o uso de acetato de medroxiprogesterona oral fora comparado ao de ant-GnRH em pacientes com síndrome dos ovários policísticos emestudo prospectivo controlado. Apesar da ausência de casos de SHO no Grupo Progestágeno, houve dois casos no Grupo Controle, sem diferença estatisticamente significante.^( 26 )^Barbosa et al., relataram prevalência de 5% de SHO em pacientes brasileiras que utilizaram 200UI por dia de FSHr associados com ant-GnRH.^( 19 )^O desencadeamento da ovulação também merece considerações. O uso de análogo ag-GnRH, no caso acetato de triptorrelina, pode ser usado tanto em protocolos de estimulação antagonista, em vez do convencional hCG,^( 25 )^quanto em protocolos de EOC com progestágeno oral com boa resposta na qualidade oocitária.^( 27 )^O ag-GnRH tem meia-vida mais curta e incidência menor de SHO quando se compara ao hCG para desencadear a ovulação. Evitar a SHO é crucial na PFO, devido ao risco de adiamento do tratamento oncológico.^( 25 )^No presente estudo, cinco em 20 casos (25%) e dois em 20 casos (10%) de SHO ocorreram, respectivamente, nos grupos progestágeno e controle, com p=0,212. O reduzido número de participantes limita uma comparação desses dados com os frequentemente relatados na literatura. Porém, essa maior percentagem de SHO poderia se relacionar ao fato de muitas pacientes serem jovens, com boa reserva ovariana e receberem dose padronizada e sem ajuste de FSHr, considerando o restrito tempo disponível para a PFO, visando ao aumento de oócitos criopreservados e, consequentemente, às chances reprodutivas. Destaca-se a ausência de casos de ascite, sendo todos classificados como SHO leve.

Outro progestágeno oral, a didrogesterona, foi comparado ao acetato de medroxiprogesterona em estudo prospectivo randomizado controlado. Não houve diferença estatisticamente significante no número de óocitos, embriões, e taxa de gravidez. Adicionalmente, a didrogesterona inibiu, efetivamente, o aumento prematuro do LH sem impactar os valores séricos da progesterona.^( 28 )^Isso justificou a inclusão tanto do uso de acetato de medroxiprogesterona quanto de didrogesterona no grupo com uso de progestágeno oral. Devido à restrita amostra por conveniência, optou-se por não fazer a separação por tipo de medicação oral na análise estatística.

Nesse contexto de boa efetividade no uso de progestágeno oral na qualidade oocitária e embrionária, Massin recomendou seu uso em relação à preservação da fertilidade não oncológica, considerando a melhoria na técnica de criopreservação embrionária e evitando a transferência imediata de embriões em um endométrio potencialmente prejudicado na receptividade endometrial.^( 27 )^

De modo semelhante, La Marca et al., reforçaram a ideia da segurança e da efetividade dos progestágenos orais durante a EOC, considerando-os boa alternativa, a fim de evitar a luteinização precoce, com bons resultados no número e na qualidade oocitária e embrionária, além do baixo risco de SHO. Apesar de também não citarem seu uso na PFO, destacaram a necessidade de mais estudos sobre os resultados reprodutivos, obstétricos e neonatais ao longo prazo nas demais situações.^( 29 )^

Entretanto, ao se considerarem pacientes com câncer de mama com receptores negativos para progestágeno, assim como aqueles com outras neoplasias, como as endometriais, nas quais o progestágeno possui efeito protetor,^( 30 )^pode-se fazer o seguinte questionamento: por que não progestágeno oral em casos selecionados de PFO? Afinal, trata-se de uma medicação que se demonstrou efetiva, com menor custo e maior conforto em relação a uma segunda medicação injetável durante a EOC, sem mecanismos de ações evidentes que poderiam piorar o prognóstico oncológico. Além disso, o curto período de uso durante a EOC também é fator motivador para seu uso.

O número reduzido de pacientes é um viés deste estudo-piloto com amostra de conveniência. Consequentemente, limita-se a capacidade de generalização dos dados. Todavia, o rigor no critério de seleção de amostra de pacientes com receptores progestagênicos negativos na avaliação imunoistoquímica sem uso de contracepção hormonal nos 3 meses que antecederam o tratamento propiciou um grupo exclusivo que justificou a realização do estudo. A homogeneidade dos grupos estudados e os resultados superiores a 80% de oócitos maduros e prontos para serem criopreservados são fatores que estimulam novos estudos, a fim de estabelecer a segurança e a efetividade de protocolos com progestágenos em pacientes oncológicas.

Outro fator limitante é a não avaliação hormonal durante os ciclos de EOC. As limitações inerentes a uma amostra de conveniência e o tempo escasso para o início de quimioterapia, a fim de não postergar o tratamento oncológico, permitiram que o início da EOC ocorresse, em alguns casos, na primeira avaliação médica, restringindo uma avaliação hormonal desde o início do ciclo. Entretanto, reforçam-se os dados clínicos e laboratoriais de um seleto grupo deste estudo.

Dessa forma, a PFO com progestágeno oral é uma opção viável, que poderia ser explorada em estudos de não inferioridade, a fim de aumentar não apenas as estratégias terapêuticas, mas elevar a experiência clínica, visando à redução de custos, fato fundamental para a ampliação de programas de preservação da fertilidade dentro da visão de um tratamento mais humanizado.

## CONCLUSÃO

O uso de progestágeno oral, apesar de necessitar de maior tempo de tratamento, é efetivo em relação ao protocolo com antagonista do hormônio liberador de gonadotrofina e oferece maior conforto com menor custo em pacientes com câncer de mama com receptores de progesterona negativos submetidas à preservação da fertilidade oncológica.
